# Cost-Effectiveness of Alternative Treatment Strategies of Subretinal Macular Hemorrhage

**DOI:** 10.3390/healthcare13131550

**Published:** 2025-06-29

**Authors:** Filippo Confalonieri, Silvia N. W. Hertzberg, Krystian Andrzej Dziedzic, Xhevat Lumi, Lyubomyr Lytvynchuk, Ljubo Znaor, Goran Petrovski, Beáta Éva Petrovski

**Affiliations:** 1 Department of Ophthalmology, Montecchi Hospital of Suzzara, via Generale Cantore 14/B, 46029 Suzzara, Italy; 2 Department of Biomedical Sciences, Humanitas University, 20072 Milan, Italy; 3 Center for Eye Research and Innovative Diagnostics, Department of Ophthalmology, Institute for Clinical Medicine, University of Oslo, Kirkeveien 166, 0450 Oslo, Norway; swalekhwa@gmail.com (S.N.W.H.); krystian.dzc@outlook.com (K.A.D.); beata.petrovski@medisin.uio.no (B.É.P.); 4 Department of Ophthalmology, Oslo University Hospital, 0450 Oslo, Norway; 5 Department of Ophthalmology, Justus-Liebig University Giessen, Eye Clinic, University Hospital Giessen and Marburg GmbH, Klinik und Poliklinik für Augenheilkunde, Friedrichstr. 18, 35392 Giessen, Germany; xhlumi@hotmail.com (X.L.); lubko_l@yahoo.co.uk (L.L.); 6 Department of Ophthalmology, University Hospital Centre, School of Medicine, University of Split, 21000 Split, Croatia; ljuboznaor@gmail.com; 7 School of medicine, UKLONetwork, University St. Kliment Ohridski-Bitola, 1 Maj bb., 7000 Bitola, North Macedonia

**Keywords:** subretinal macular hemorrhage (SRMH), cost-effectiveness in ophthalmology, anti-VEGF therapy, tPA, pars plana vitrectomy, best-corrected visual acuity (BCVA)

## Abstract

**Purpose:** To evaluate the cost-effectiveness of alternative treatment strategies for subretinal macular hemorrhage (SRMH), a condition often associated with neovascular age-related macular degeneration (AMD) and other retinal vascular disorders, leading to severe visual impairment. **Methods:** A retrospective cross-sectional study conducted at Oslo University Hospital assessed the cost and utility of various SRMH treatment modalities. These included intravitreal anti-VEGF monotherapy, intravitreal tissue plasminogen activator (tPA) with gas displacement (alone and in combination with anti-VEGF), and pars plana vitrectomy (PPV) with subretinal tPA and gas displacement (with and without anti-VEGF). Costs were analyzed from a healthcare perspective, encompassing direct and indirect costs. Effectiveness was measured using median best-corrected visual acuity (BCVA) improvements. Sensitivity analyses were performed to account for complications and variations in follow-up. **Results:** Anti-VEGF monotherapy was the most cost-effective treatment, with the lowest cost per unit of BCVA improvement (NOK 44,717) in outpatient settings. Intravitreal tPA with gas displacement emerged as a cost-effective alternative but exhibited higher costs when combined with anti-VEGF or performed as an inpatient procedure. PPV with subretinal tPA and gas displacement, with or without anti-VEGF, was the least cost-effective modality, particularly in inpatient settings. Sensitivity analyses indicated that anti-VEGF therapy remained cost-effective even with increased follow-up requirements and complications, while tPA-based therapies required significant BCVA improvements to match anti-VEGF’s cost–utility. **Conclusions:** Outpatient intravitreal anti-VEGF monotherapy followed by tPA with gas displacement are the most cost-effective strategies for SRMH management. Subretinal tPA-based treatments are associated with higher costs and limited economic viability, highlighting the importance of tailored treatment selection. These findings support strategic resource allocation in managing SRMH while optimizing patient outcomes.

## 1. Introduction

Subretinal macular hemorrhage (SRMH) is a serious ophthalmic condition that occurs when blood accumulates beneath the retina, specifically in the macula [1]. It often results in severe visual impairment, as the macular region is responsible for sharp central vision, hence affecting activities like reading, driving, and recognizing faces. SRMH is most commonly associated with various retinal vascular conditions such as neovascular age-related macular degeneration (AMD), polypoidal choroidal vasculopathy (PCV), retinal vein occlusion, and following trauma or other retinal disorders [2]. Undiagnosed or untreated hemorrhages pose a significant risk to vision, as they can damage the delicate structures of the retina, leading to irreversible visual impairment that may potentially lead to blindness. Early intervention is crucial to prevent severe outcomes [3,4].

SRMHs are observed frequently in older adults with advanced AMD, a leading cause of blindness in the aging population. The global prevalence of AMD is estimated at 8.7%, while in Europe alone, the number of advanced AMD patients is expected to rise to 4.8 million by 2040 [5,6,7,8]. Among patients with AMD, up to 17% are likely to develop subretinal hemorrhage, and with an increasing aging population, the incidence of hemorrhagic conditions is expected to rise dramatically [2,9].

The management of SRMH varies and is mostly dependent on the patient’s overall condition, the size, location, and the cause of the hemorrhage. Current treatment modalities for SRMH focus on minimizing damage to the macula and preserving vision. Modalities that have gained popularity and are frequently used are anti-vascular endothelial growth factor (anti-VEGF) injections such as ranibizumab, aflibercept, or bevacizumab. These injections are characterized by multiple follow-ups over a period and help stabilize or improve visual outcomes by reducing abnormal blood vessel growth as well as leakage [10,11]. Other treatment modalities include pneumatic displacement with or without tissue plasminogen activator (tPA), employed to displace the subretinal blood. This method is sometimes combined with a vitrectomy, where the vitreous gel is removed to allow better surgical access. For long-standing or particularly large hemorrhages, subretinal tPA injections during vitrectomy may also be used to dissolve the blood clot [12,13]. The effect of tPA combined with anti-VEGF has also been reported [14].

Despite the availability of these treatment strategies, there remains a lack of consensus on the most effective approach for SRMH, with variations in clinical outcomes based on the patient’s condition, the extent of the hemorrhage, and treatment choice [11,14]. In addition, the economic burden of SRMH can be profound, driven by direct healthcare costs (e.g., costly interventions), indirect costs (such as loss of productivity), and the overall reduced quality of life for patients. These costs are expected to further stretch healthcare resources, especially within the developed countries that have higher life expectancy and an increasingly aging population. Despite the extensive study of the clinical management of SRMH, economic evaluation in this area remains limited. Most existing economic evaluations in ophthalmology have largely focused on anti-VEGF therapy’s cost–utility for AMD or surgical approaches, such as vitrectomy, with models demonstrating variable incremental cost-effectiveness ratios [15,16,17,18]. These models not only offer a framework to understand the potential cost-effectiveness of SRMH interventions but also provide insights into resource allocation and the outcome benefit of sustained vision preservation in AMD. With a notable gap regarding economic modeling for SRMH-specific intervention in the literature, an economic evaluation is thus warranted to facilitate strategic decision making in resource allocation and priority settings, reimbursement decisions, as well as equity and treatment access [19,20].

This study aims to perform a cost–utility analysis of SRMH treatment options, including anti-VEGF therapy, intravitreal tissue plasminogen activator (tPA) injections, and pars plana vitrectomy (PPV), either as a monotherapy or combination. The purpose is to evaluate the cost-effectiveness of alternative treatment strategies for SRMH, which is often associated with neovascular AMD and other retinal vascular disorders, leading to severe visual impairment and an economic burden on healthcare systems.

## 2. Materials and Methods

This study was based on a retrospective cross-sectional study conducted at Oslo University Hospital (OUH), evaluating the efficacy of SRMH alternative treatment modalities used. Further detailed data information is explained in the paper elsewhere. The study was approved by the Privacy Officer at OUH under reference number 23/12472.

The aim of this study was to assess the cost-effectiveness of the treatment options utilized for SRMH. Specific selection criteria were applied when extracting data for the current analysis. These included a confirmed diagnosis of SRMH secondary to neovascular AMD and the availability of complete clinical and visual acuity data. Patients with significant ocular comorbidities or incomplete records were excluded to reduce potential confounding. Patients in all treatment groups had similar baseline tests and post-treatment best corrected visual acuity (BCVA) measures. The alternative treatment options assessed in this study include intravitreal anti-VEGF monotherapy injections, intravitreal tPA with intravitreal gas displacement, intravitreal tPA with gas displacement combined with anti-VEGF, and PPV with subretinal tPA with gas displacement with and without intravitreal anti-VEGF injection. The BCVA results are used as the median value of each option, which were directly derived from patient-level data at 1-month follow-up for each intervention group. The medians were used as deterministic inputs in the economic model; hence, they were not subject to statistical inference. Notably, baseline comparability was reviewed descriptively, and uncertainty around BCVA was explored through sensitivity analyses.

### 2.1. Cost

The cost of each alternative option was assessed from the healthcare perspective and comprises direct and indirect medical costs. This includes procedure costs, drug costs, follow-up costs, personnel costs, hospitalization costs, patient transportation costs, and time spent on treatment costs (Table 1). The diagnostic costs were excluded in the calculations, as all patients underwent the tests irrespective of the condition.

### 2.2. Calculations

The cost estimation for each procedure was calculated using the disease diagnostic group (DRG) weights multiplied by the Norwegian 2024 unit price, which represents the relatively true costs of activities [21]. The drug costs, including those for aflibercept, ranibizumab, bevacizumab, and dexamethasone, were collected from the Norwegian medicine association, and an estimation without value added tax was added and averaged before being added to the anti-VEGF procedure costs. Follow-up for each procedure was multiplied by twice the transportation cost to cover the return journey. The time spent on the journey and at the treatment center (assumed to range between 1.5 and 2 h for outpatient and 24 h for inpatient cases) was accumulated and multiplied by the recorded cost per hour of the patient’s time. These costs were aggregated to the procedure costs of each treatment option. Furthermore, an admission cost was added to inpatient treatments to estimate the total costs.

The follow-up time did not indicate the complications of the treatment options herein. However, several studies show varying percentage rates of occurrence after treatment (Table 2). Therefore, a range of known complications for different alternative options have been assessed in a series of sensitivity analyses [22,23,24,25].

The cost-effectiveness of the alternative treatment for SRMH was estimated by dividing the total cost of each treatment option by the relevant median BCVA value. This was repeated for inpatient options, and results were recorded differently.

## 3. Results

**Figure 1:**
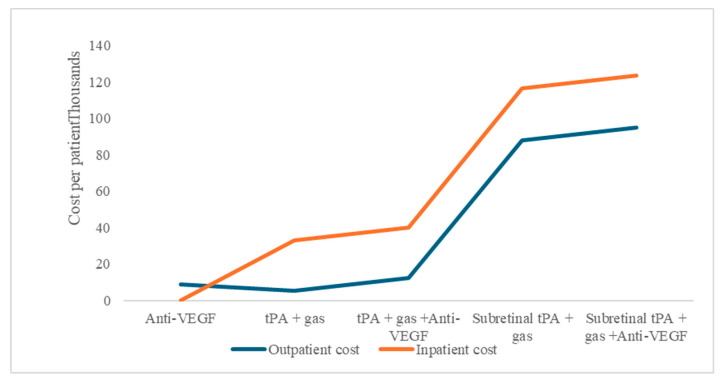
Subretinal macular hemorrhage treatment option average costs per patient, as outpatient or inpatient procedures.

The study aim was to perform a cost–utility analysis of alternative treatment options used in managing SRMH, which includes anti-VEGF therapy, intravitreal tissue plasminogen activator (tPA) injections, and PPV, either as a monotherapy or combination. These treatment options for SRMH indicate different economic resource use. Comparatively, for procedures that can be performed as outpatient or inpatient, there are consistently higher costs for admitted patients in our model under the base scenario (Figure 1).

The use of anti-VEGF shows good value in the monotherapy group, with a lower cost per unit of BCVA improvement of NOK 44,717 compared to the alternative options at baseline. In addition, outpatient treatments, tPA with gas displacement alone, provide better resource use compared to anti-VEGF (Table 3) or tPA with gas displacement combined with anti-VEGF compared to subretinal alternatives. However, the lower cost of tPA with the gas displacement option does not amount to better cost-effectiveness compared to anti-VEGF monotherapy. For inpatients, the combination of tPA, gas, and anti-VEGF is more cost-effective than tPA and gas alone, contrary to the outpatient situation, where the combination is almost double the cost per unit of BCVA improvements (Table 3).

**Figure 2:**
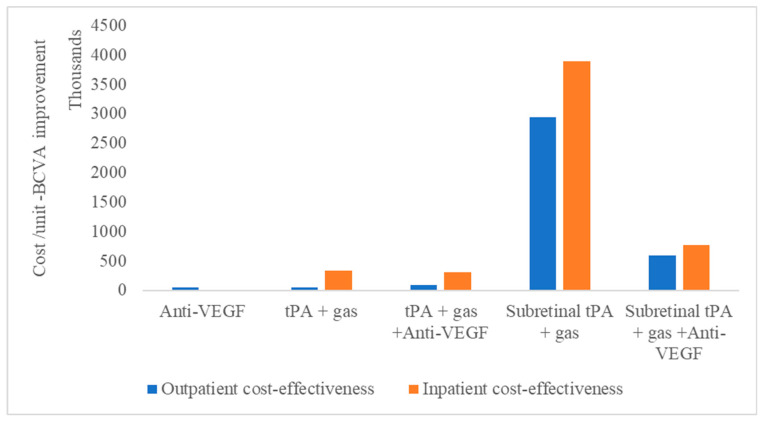
Cost-effectiveness of subretinal macular hemorrhage treatment options as outpatient or inpatient procedures.

Meanwhile, for vitreoretinal procedures, subretinal tPA with gas displacement combined with anti-VEGF is shown to be more cost-effective than without anti-VEGF (Figure 2).

This is irrespective of whether the treatment is performed as an outpatient or inpatient procedure. Therefore, the least cost-effective treatment modality, irrespective of whether it is outpatient or inpatient, remains tPA with gas displacement alone.

### Sensitivity Analysis

**Figure 3:**
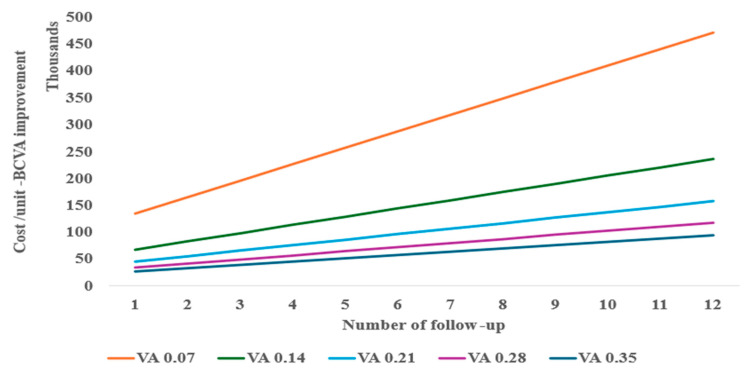
Two-way sensitivity of follow-up and BCVA for anti-VEGF cost-effectiveness.

In a one-way and two-way sensitivity analysis considering various follow-ups needed for re-injection, the anti-VEGF monotherapy required at least two follow-ups (Figure 3) to be similar in cost per unit of BCVA improvement to tPA with gas displacement alone at baseline performed as an outpatient procedure. A decrease in BCVA from the baseline of 0.21 is shown to significantly reduce the cost-effectiveness of the anti-VEGF monotherapy treatment (Figure 3).

**Figure 4:**
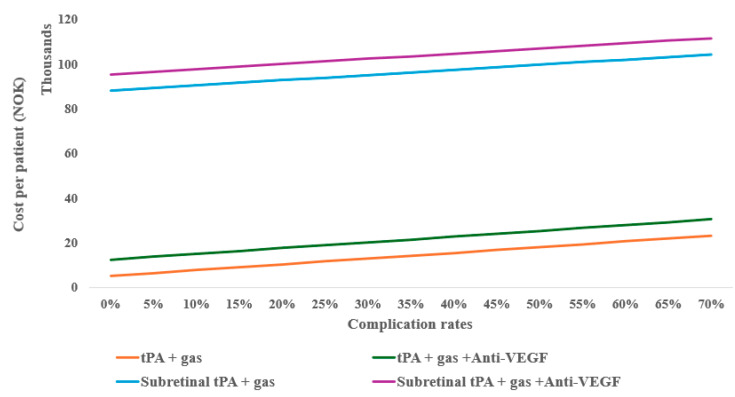
One-way sensitivity of average outpatient costs on complication rates of subretinal alternative treatments.

However, considering the possible complications of the treatments (e.g., 25%), the monotherapy anti-VEGF can still be cost-effective with up to eight follow-ups compared to tPA with gas displacement alone, as well as at 10% complication for tPA with gas displacement combined with anti-VEGF (Figure 4). When compared to inpatient treatments, anti-VEGF remained the most cost-effective, even with up to twelve follow-ups.

**Figure 5:**
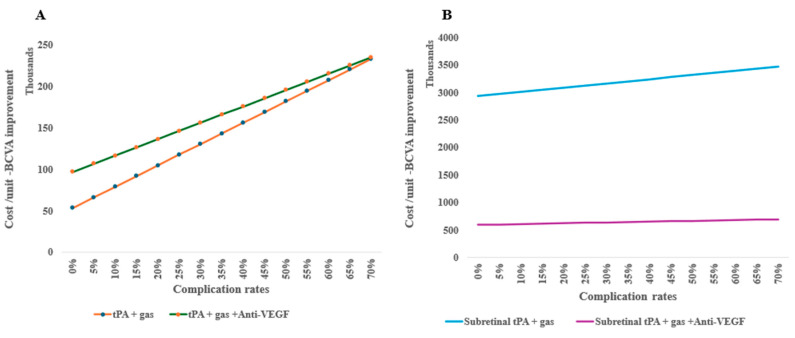
One-way sensitivity of costs per unit of BCVA improvement on complication rates of subretinal alternative treatments: (**A**) intravitreal treatments and (**B**) surgical therapy in an outpatient setting.

Treatment complications are shown to increase the cost per unit of BCVA improvements, for example, tPA and gas alone indicate escalating costs per unit more as the risk of complication increases when combined with anti-VEGF (Figure 5A). In surgical treatments, subretinal tPA combined with gas and anti-VEGF retains the cost-effectiveness irrespective of the complication rates (Figure 5B).

**Figure 6:**
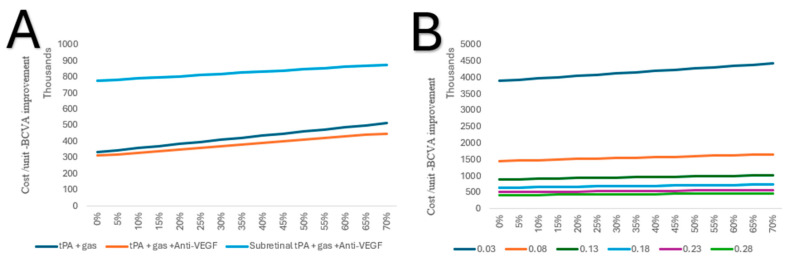
(**A**) One-way sensitivity of costs per unit of BCVA improvement on complication rates of the alternative treatments in an inpatient setting. (**B**) Two-way sensitivity analysis of costs per unit of BCVA improvement on complication rates of subretinal tPA and gas in an inpatient setting.

In a one-way sensitivity analysis, tPA combined with gas and anti-VEGF, if conducted in an inpatient setting, continues to be the lowest cost per unit of BCVA improvement, irrespective of the rate of complications. As for subretinal, tPA and gas combined with anti-VEGF is high in cost per unit of BCVA; however, the increase is lower with each rate of complication (Figure 6A). Due to the high cost per unit of subretinal tPA and gas alone, it was excluded from further comparison. However, in a two-way sensitivity analysis, it was shown that the improvement changes from baseline (0.03), with 0.05, significantly decreased the cost per unit of BCVA (Figure 6B).

## 4. Discussion

Economic evaluations play an increasingly critical role in the assessment of healthcare interventions, as healthcare systems worldwide face growing pressure to allocate limited resources efficiently [26]. This trend is particularly evident in the field of ophthalmology, where a wide range of innovative but often costly diagnostic tools, surgical techniques, and pharmacological treatments are being developed and adopted [27]. Conducting robust health economic analyses—such as cost–utility, cost-effectiveness, and budget impact studies—has become essential to inform policy decisions, prioritize funding, and guide clinical practice. These evaluations not only help determine the value of new and existing ophthalmic interventions but also support evidence-based strategies that maximize patient outcomes while ensuring sustainable healthcare delivery.

This study’s aim was to evaluate the cost-effectiveness of various treatment modalities available for SRMH. The efficiency of such treatments has been shown in multiple studies, but due to the lack of conclusive optimal treatment options, it is up to the doctor’s discretion and the patient’s condition to facilitate the treatment of choice [11,12].

Anti-VEGF monotherapy has been shown to be more effective for small-sized SRMHs compared to surgical treatment approaches. In this study, we found that the cost of such an approach is lower and more cost-effective compared to other alternative approaches under the baseline scenario. At 12 months, however, anti-VEGF monotherapy has been shown to provide similar effects to those of the surgical modalities in managing large-sized hemorrhages [24]. Hence, in the sensitivity analysis, we show varying follow-up re-injections at 12 months, finding the costs to still be lower (63% or 65%), even with monthly re-injections compared to vitrectomy with subretinal tPA and gas displacement alone or with anti-VEGF at baseline (no complications). Previous studies with follow-up periods extending up to one year have reported mean follow-up durations of approximately 1.4 to 3 years. Under these baseline assumptions, anti-VEGF monotherapy in our model proved to be more cost-effective than all other options [28,29,30,31,32].

The tPA with gas displacement alone was more cost-effective as an outpatient procedure compared to tPA with gas displacement combined with anti-VEGF. However, for inpatients, the latter was more cost-effective, presenting lower costs per BCVA unit compared to those without anti-VEGF. In a two-way sensitivity analysis, a 0.15 additional improvement in median BCVA could facilitate better cost-effectiveness (approximately 83%) at baseline. Considering the complication of tPA with gas displacement alone, similar cost-effectiveness to that with anti-VEGF can be achieved at a greater than 70% rate (>0.15 BCVA change).

Comparing whether tPA with gas displacement combined with anti-VEGF should be performed as inpatient or outpatient care, the result within this model’s limit shows outpatient care to be more cost-effective, with almost three times lower costs per unit BCVA improvement compared to inpatient care. However, under outpatient care, the recovery time is not factored in, so this cost may be higher when added to the total cost, thus reducing the cost-effectiveness.

Our findings indicate that intravitreal anti-VEGF monotherapy and tPA with gas displacement, when performed as an outpatient procedure, offer the most cost-effective treatment option for SRMH management. Anti-VEGF therapy, a cornerstone in managing neovascular AMD and associated hemorrhages, appears to demonstrate consistently favorable cost–utility metrics, reflecting its established efficacy in stabilizing or improving visual outcomes with relatively low associated costs. Similarly, tPA with gas displacement as an outpatient procedure emerged as a viable alternative (Figure 2 and Figure 4), offering a favorable balance between cost and effectiveness for patients with moderate to severe hemorrhages.

The patients who had PPV and subretinal tPA with gas displacement experienced the lowest median BCVA; meanwhile, the cost per unit was almost five times that when anti-VEGF was included (Figure 2). This result shows how this treatment alternative for SRMH is not economically viable, whether it is performed as outpatient or inpatient care, and especially where additional intravitreal anti-VEGF injections can be used for a better outcome. Other studies have also reported the diminishing visual acuity achieved over time with the use of subretinal tPA combined with gas and suggested continuous use of anti-VEGF postoperatively to sustain visual gains [33] Hence, a two-way sensitivity analysis indicates that PPV with subretinal tPA with gas displacement alone needs to achieve a higher median BCVA to match other alternative treatment options.

The literature on treatment complication rates varies widely, and most studies performed have a short follow-up time. In addition, baseline characteristics differ across studies, making direct comparisons impossible. For instance, studies that focused on AMD and PCV exhibited higher rates of complications [34,35]. Hence, our sensitivity analysis provides for the range, noting this as an outlier for indicating probable true complication costs. From our observation, as well as our review of the literature, lower rates of complications are reported in recent studies than former ones. However, in this case, we considered both rates for the treatment option, as recorded [14].

Various studies have shown how expertise can facilitate better improvements with minimal avoidable complications and the association of preoperative VA with improvements in visual prognosis [13,36,37]. Therefore, physician experience remains vital, especially for invasive procedures. More so, the knowledge of the VA threshold value for determining the choice of alternating treatment, as well as the associated patient anatomic characteristics, will sustain the cost-effectiveness of these options.

This study has several limitations. First, the short follow-up limits the ability to determine the long-term complications, further improvements, or decline in BCVA of the alternative treatments that may occur with time. Second, reliance on retrospective data where treatment patient groups were non-randomized could introduce selection bias and confounding, as BCVA outcomes may have been affected by unmeasured patient characteristics in either of the groups. Third, the model was based on real-world cost data from a Norwegian healthcare setting; hence, it may limit the generalizability to other contexts or countries. Fourth, in line with the economic evaluation guidelines, clinical outcomes were not subjected to formal hypothesis testing or confidence interval estimations, prompting a series of one-way and two-way sensitivity analyses. These parameter inputs were thoroughly assessed, and the analyses demonstrated steady robustness of the conclusions across plausible ranges. Furthermore, the statistical analysis does not account for potential confounding factors or interactions. Despite these limitations, this study not only provides an important initial economic perspective on the management of SRMH but also highlights the necessity for prospective cost–utility evaluations with longer-term outcomes.

## 5. Conclusions

The aim of this study was to estimate the cost-effectiveness of alternative surgical and pharmacological treatments for submacular retinal hemorrhage (SRMH). To date, none of these treatment options have been shown to be clinically superior to the others in terms of functional outcomes or safety. However, our findings highlight significant differences in cost-effectiveness among the available strategies. Specifically, we determined that pars plana vitrectomy (PPV) combined with subretinal administration of tissue plasminogen activator (tPA) and gas was the least cost-effective option. Given its higher expense and lack of clear clinical advantage, this approach may not be justifiable in most settings, especially where more straightforward treatments—such as anti-VEGF injections—remain a feasible option.

Additionally, our analysis reinforces that anti-VEGF monotherapy is the most cost-effective intervention overall. It not only reduces healthcare expenditures but also provides a practical, readily accessible alternative with good visual outcomes for appropriately selected patients. Finally, we found that intravitreal tPA-based strategies offer a more cost-effective profile than subretinal tPA administration. This suggests that intravitreal tPA combinations may be preferable for most patients with SRMH, reserving subretinal tPA administration for specific, carefully selected cases where its unique benefits could outweigh its higher cost.

## Figures and Tables

**Table 1:** Unit costs and data sources.

Treatment Option	Unit Costs (NOK)	Source
Intravitreal anti-VEGF drugs		(Drug catalog, 2024)
Avastin 1 × 4 mL	384	
Eylea 40 mg/mL	3568	
Ozurdex 700 mg	10,885	
Lucentis 10 mg/mL	3402	
Tissue plasminogen activator (tPA) and gas	3005	(OUH)
Pars plana vitrectomy	83,545	(Unit cost database, 2024)
Scleral buckling	13,532	(Financing, 2024)
Phacoemulsification	12,592	(Financing, 2024)
Intravitreal injection	3553	(Financing, 2024)
Other outpatient examination and treatment of eye conditions with specified measures	1829	(Financing, 2024; Unit cost database, 2024)
Patient time cost/hour	368	(Unit cost database, 2024)
Transport cost/one-way	794	(Unit cost database, 2024)
General admission	19788	(Unit cost database, 2024)

**Table 2:** Occurrence rate of treatment option’s possible complications and relevant best-corrected visual acuity median values.

Treatment Option	Complication	Occurrence Rate	BCVA Median
Intravitreal anti-VEGF monotherapy	Insignificant	0	0.21
Intravitreal tPA with gas displacement	Recurrent sub-macular hemorrhage	0–27%	0.1
	Vitreous hemorrhage	0–45%	
	Retinal detachment	0–7%	
Intravitreal tPA with gas displacement combined with anti-VEGF	Recurrent sub-macular hemorrhage	0–13.6%	0.13
	Vitreous hemorrhage	0–43%	
	Retinal detachment	0.5%	
PPV with subretinal tPA and gas displacement	Recurrent sub-macular hemorrhage	0–27%	0.03
	Vitreous hemorrhage	0–67%	
	Retinal detachment	0–11.8%	
	Cataract	0–11.5%	
PPV with subretinal tPA and gas displacement combined with anti-VEGF	Recurrent sub-macular hemorrhage	0–20%	0.16
	Vitreous hemorrhage	0–38%	
	Cataract	0–11.5%	

**Table 3:** Total cost per patient and the cost-effectiveness of alternative subretinal macular hemorrhage treatments.

Treatment Option	Outpatient Cost (NOK)	Outpatient Cost/Unit of BCVA Improvement	Inpatient Cost (NOK)	Inpatient Cost/Unit of BCVA Improvement
Anti-VEGF	9391	44,717	0	0
Intravitreal tPA + gas	5329	53,285	33,210	332,103
Intravitreal tPA + gas + Anti-VEGF	12,579	96,762	40,461	311,237
Subretinal tPA + gas	88,137	2,937,909	116,755	3,891,827
Subretinal tPA + gas + Anti-VEGF	95,388	596,174	124,005	775,034

## Data Availability

Data can be requested on reasonable requests from the authors.
